# Transcribing historical Canadian weather data

**DOI:** 10.1038/s41597-025-06036-y

**Published:** 2026-04-29

**Authors:** Victoria Slonosky, Rachel Black, Lori Podolsky, Xiaolan Wang, Vincent Cheng

**Affiliations:** 1 Open Data Rescue, Saint-Lambert, Canada; 2 Affiliate Researcher, Tomlinson Lab, McGill University, Montreal, Canada; 3 Nova Scotia Provincial Archives, Halifax, Canada; 4 Texas A&M Corpus Christi, Corpus Christi, USA; 5 Climate Research Division, Environment and Climate Change Canada, Toronto, Canada; 6 Environment and Climate Change Canada, Suite 200, 2474 Arbutus Rd, Victoria, BC, V8N 1V8 Canada

**Keywords:** Atmospheric science, Climate change

## Abstract

Historical weather journals from across Canada, spanning 1768–1884, have been transcribed from handwritten records into machine readable formats. The NORTHERN (Nineteenth-century Overseas Records Transcribed for Historical Environmental Reconstruction in the North) project transcribed nearly 2 million weather observations from 46 locations. The original documents are in archives outside Canada. The two principal archives investigated for historical Canadian weather are the United States’ National Administration and Records Archives (NARA) and the United Kingdom’s Meteorological Office (UKMO) Library and Archives. Some observations were also located in the United States’ National Centers for Environmental Information (NCEI) “Forts” dataset. Observers recorded from three to twenty weather variables, in most cases two or three times daily. Validation procedures are carried out with export files produced in both the original format and in modern units. Observations of pressure, temperature, precipitation, snow depth, cloud cover, cloud type, wind direction and wind force are transcribed along with detailed descriptions of events including fires, floods, ice formation and break up, storms and other weather phenomena. The value of these data lies in their detailed observations of sub-daily weather together with descriptive observation of disruptive or extreme weather events. These data will be used to expand knowledge of Canada’s climate variability and extreme values for three centuries and to improve global reanalysis data products.

## Background & Summary

There exist millions of historical weather observations from Canadian locations in paper format in archives in Canada and around the world^1^. These historical records are of immense importance in understanding long term climate change^2–4^, climatic extremes and high impact weather events^5,6^, and in constraining and validating climate models^7^. Historical observations such as those presented in this paper are collected into international data collections^8,9^ and are used as the input data to drive the numerical weather prediction model which produce reanalysis data, such as the Twentieth Century Reanalysis Project (20CR)^10,11^. The high-quality, multi-variable and sub-daily observations and the reanalysis models they enable are particularly vital for furthering our understanding of rare, disruptive or high impact events which caused local, regional and global social disruption, such as the Year with a Summer of 1816, following the Tambora eruption of 1815^12^.

Here we present a collection of digitally transcribed (machine readable) and quality-controlled historical weather observations for the 18^th^ and 19^th^ centuries in Canada, concentrating on the period before the founding of the Meteorological Service of Canada (MSC) in 1873. These observations are housed in archives outside present-day Canadian territory. We note here that the territory that is now Canadian underwent considerable evolution over the past three centuries. In the early nineteenth century, Canada consisted of British colonies known as “British North America” in southern Ontario, Quebec, New Brunswick, Nova Scotia, Prince Edward Island and Newfoundland. The Hudson Bay Company had been granted the land covered by the drainage basin of the Hudson Bay, a territory then known as Rupert’s Land. At Confederation in 1867, the colonies of Ontario, Quebec, New Brunswick and Nova Scotia formed the country of Canada. The territory of Rupert’s Land became attached to Canada in 1870, along with the area near the Red River Settlement (Winnipeg). British Columbia was formed in 1871, Prince Edward Island joined Canada in 1873, and Newfoundland in 1949. Thus, many of the locations now in Canada were administered by different entities during the period of interest here, and many meteorological records can be found in non-Canadian archives.

The main source of these observations  with 32 records, is the United States National Archives and Records Administration (NARA), specifically those housed in the NARA M1958 collection, consisting of eight rolls of microfilms of weather records from 19^th^ century sources outside the contiguous United States^13^. The microfilms were produced by NARA in 2005. We designate this source “NARA M1958”. Two additional records were found in the US National Centres for Environmental Information (NCEI) “Forts” Dataset^14,15^ designated here “NCEI-Forts.”

**Fig. 1:**
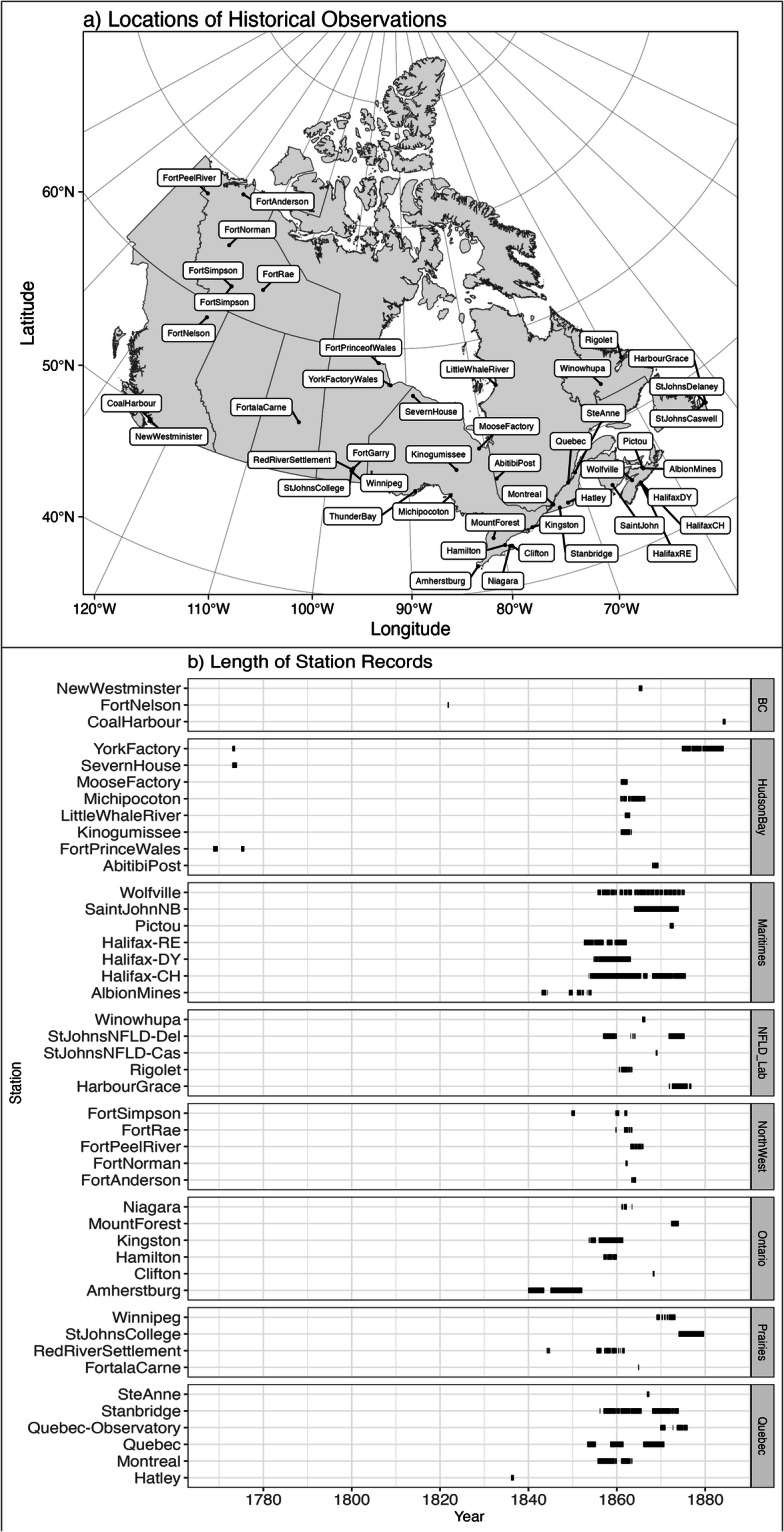
(**a**) Station Locations, (**b**) timelines of the period covered by each station.

Locations where existing information is sparse, particularly in the Canadian north and north-west^1,5^ were preferentially selected. The two determinants of areas of existing observational scarcity and record longevity were often mutually exclusive, as longer, more stable observation recording locations tended to be in more southern areas, with some exceptions (Fig. 1). Considerable effort was expended on the short, fragmentary, and often difficult to decipher records of the Canadian North-West. These image files were uploaded into an online app where values were transcribed directly into a relational database^16,17^. Efforts are made to transcribe as much as possible of the original documents and all of the information for each site in order to conserve our scientific heritage, meteorological and climatological intra-variable coherence, data validation and data traceability. All the information from Canadian stations in the NARA^13^ and Forts^14^ archives were extracted. Some of the information from the UKMO Royal Engineers and Army Medical Department were transcribed in an earlier project (see below). We believe we have extracted the main sites and information for Canadian stations from the UKMO official British Army archives; however, further Canadian observations may remain in other UKMO collections.

Earlier projects in 2017 and 2019 located the records and identified the observational data and structure in the NARA and NCEI archives. A pilot project (*Transcribing Historical Canadian Climate Records: the Red River Settlement and York Factory Records*) ran from January to March 2020, where observations from the stations of York Factory^13^ (1874–1884; 204,028 data points) and the Red River Settlement^13^ (1844, 1855–1861; 31,798 data points) were digitized by 16 transcribers. Following the success of this project, a more substantial project (*Transcribing Historical Canadian Weather Data: The Smithsonian Records*), renewed twice in 2022 and 2023, was organized to expand on and transcribed observations from the rest of the Canadian stations in the NARA^13^ and Forts^14^ datasets. This amounted to 496,578 data points transcribed by 12 transcribers in the first year (2021–2022) and 744,523 data entries by 11 transcribers the second year. As the project entered its third year, most of the observations from the NARA and NCEI sources had been transcribed, and so meteorological records from the United Kingdom were investigated. Twelve records were found in the United Kingdom’s Meteorological Office (UKMO) archives. Seven transcribers entered 482,829 data points in the final year of the project.

The transcribed values are verified, validated and transformed into modern international units and exported into machine readable text files. Here we use “verify” to denote a process to check that the values transcribed are faithful to the original observations recorded by the historical observers. We use “validate” to indicate the transcribed values are within the normal ranges for the meteorological variable observed. The two process are sometimes in conflict, as when the original observer transposed digits. In these cases, the original observation is flagged and, if possible (e.g. the observer wrote “15” instead of “51” for a summertime temperature registered in Fahrenheit degrees), the transformed value is altered in the validation stage. All changes are automatically recorded. The initial export is a csv format which reproduces as faithfully as possible the layout of the original register pages. Other export formats are the Station Exchange Format (SEF)^18–20^ and the NCEI recommended csv format^21^.

Many of the historical observations transcribed are weather records kept by volunteer weather observers and transmitted to the Smithsonian Institute as part of the volunteer Smithsonian Meteorological Project organized and maintained by Joseph Henry^22–24^. Although originating in the United States, one of Henry’s goals was the understanding and predictions of storms, and thus observations were collected from across North America. The project began in 1849. The U.S. Civil War of 1861–1865 severely disrupted the observation network in the United States^24^, although it was a peak period of observations in Canada (Fig. 1b). After the Civil War, responsibility for communications was taken up by the US Signal Service.

**Fig. 2:**
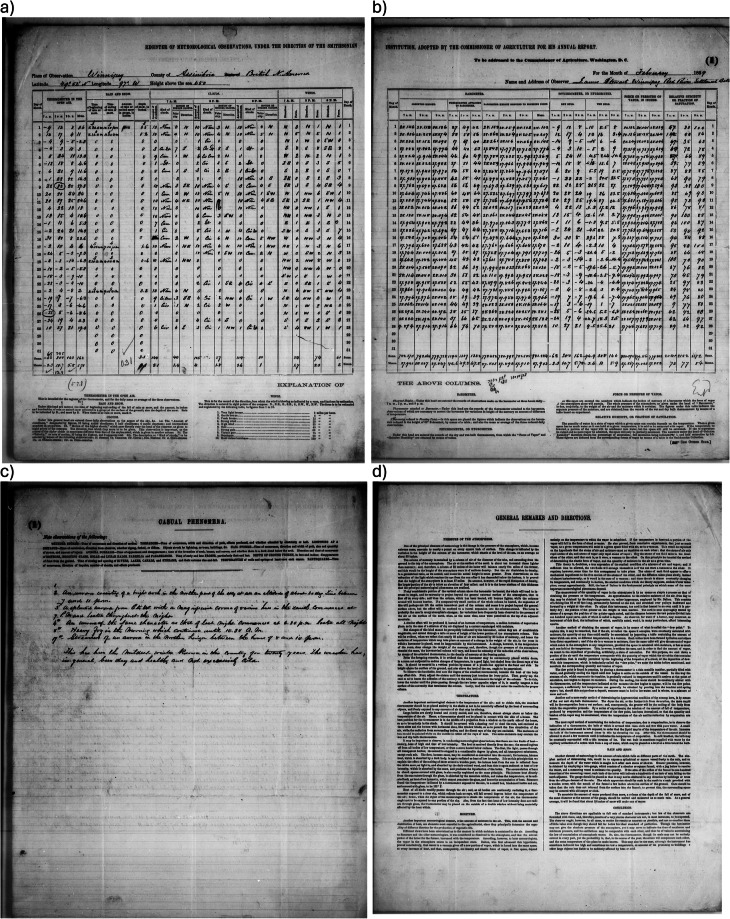
Examples of pages. (**a–c**) Clean and legible pages for Winnipeg, pages a) OBS-L (Observations-Left), (**b**) OBS-R (Observations-Right), (**c**) CP (Casual Phenomena), (**d**) Instructions to observers^13^.

Printed forms with a detailed set of instructions on how to observe and record weather and meteorological phenomena were sent to observers to be filled out and returned at the end of each month (see Fig. 2). Many of the volunteer weather observers had already been engaged in recording the weather and in some cases other copies of their observations exist in local or other international collections. The value of the Smithsonian collection is that the participants were requested to observe a standard set of variables at specific observing times, usually 7AM, 2PM and 9PM local time, at stations across the continent. This gives the set of observations standard observing practices, a set of commonly observed variables and regularized register forms. These aspects increase confidence in the accuracy of the observations and make it possible to design a web-based transcription process.

**Fig. 3:**
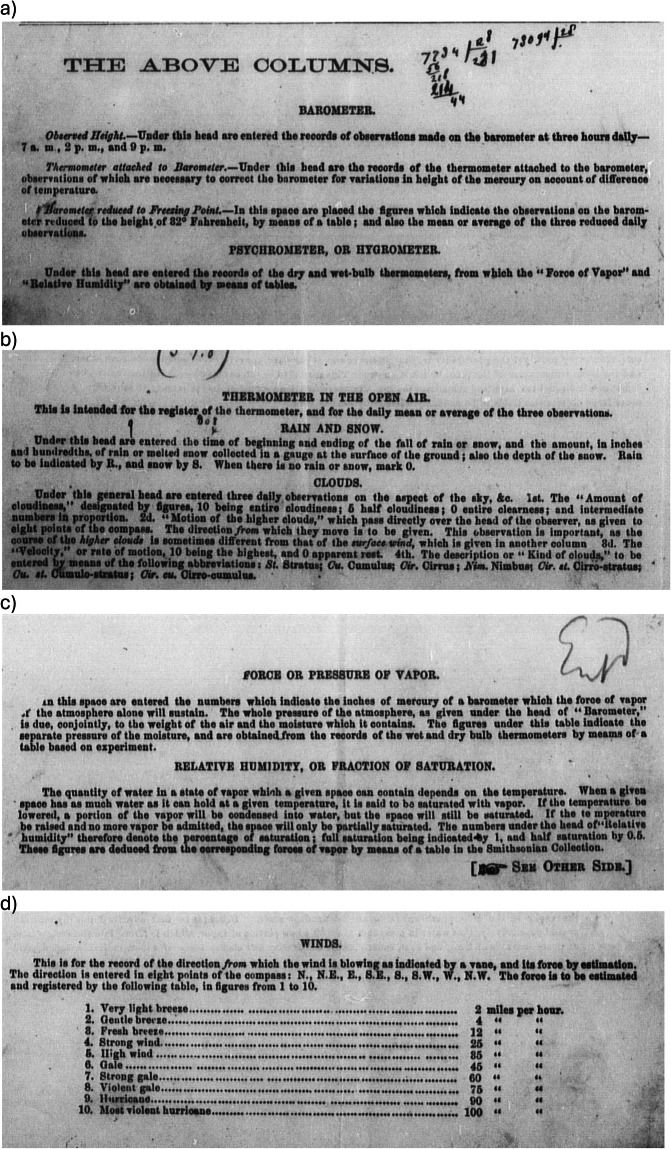
Examples of instructions and guidelines (**a**) close-up of bottom left from Fig. 2a, (**b**) close up of bottom right from Fig. 2a, (**c**) close-up of bottom left from Fig. 2b, (**d**) close up of bottom right from Fig. 2b^13^.

The consideration of how to gauge the trustworthiness of historical observations is complex^25^. Instructions to observers include both specific instructions of daily readings (Fig. 3) and more general remarks on the placement of instruments. Barometers “may be conveniently placed within doors, in a room not subjected to sudden changes of temperature, in a good light but shaded from the direct rays of the sun”^26^. Thermometers “should not be placed in contact with the side of a house. The best position for the thermometer is in the middle of a projection from a window on the north side of the house, so as to be entirely in the shade”^26^.

Other records in these archival collections include documents from both individual observers and from organized weather collection efforts by the United States' Surgeon General’s (USSG) Office. After the Smithsonian ended direct involvement in the volunteer weather project in 1870, the United States Signal Service (USSS) continued to collect weather observations from volunteer observers^13^. Some records from current Canadian territory can also be found in the United States' National Centre for Environmental Information (NCEI), in the “Forts” data collection^14^.

Weather registers and journals are also found in the archives of the UKMO^27,28^. Among these are the observations kept by the Royal Engineers and later the Army Medical Department^28–37^. The Royal Engineers (RE) also observed the weather at specified local times and according to systematic instructions and on pre-printed register forms^38^.

## Methods

**Fig. 4:**
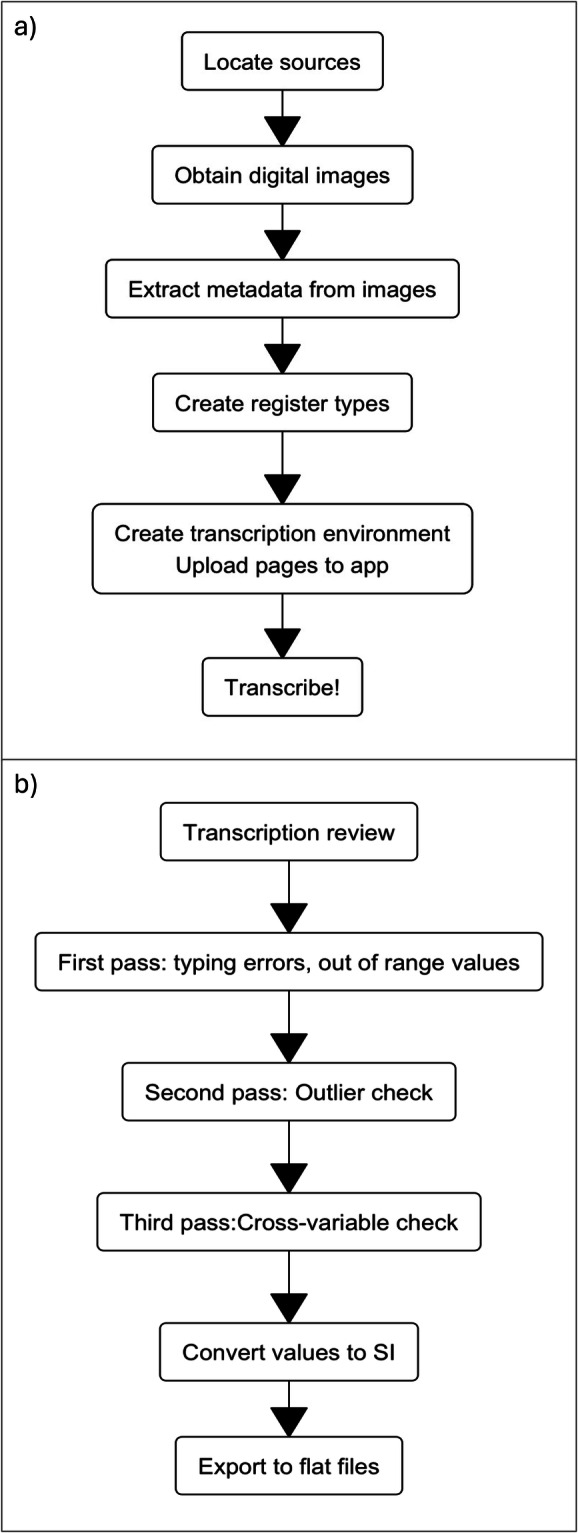
The pre- and post-processing climatic data extraction procedures. (**a**) Pre-transcription processing steps focus on obtaining and cataloguing images of the weather observations and formatting the transcription app. (**b**) Once the data is transcribed, it is verified using various techniques and converted to standard modern units.

The procedures used to recover the historical weather and climate data can be divided into three main parts. The first is the pre-transcription processing, which includes locating the observations, obtaining digital image files, processing the image files and configuring the app for data transcription (Fig. 4a). The second part is the actual transcription process. The third part is the post-transcription processing of the now machine-readable weather observations (Fig. 4b).

A traceable transcription and validation process is critical to maintaining transparency in data records. Here, traceability starts with the maintaining of a connection to the original archival record source through the medium of the digital image file of the original meteorological observations. The code for the transcription app can be found on the GitHub platform^39^.

### The image files

**Fig. 5:**
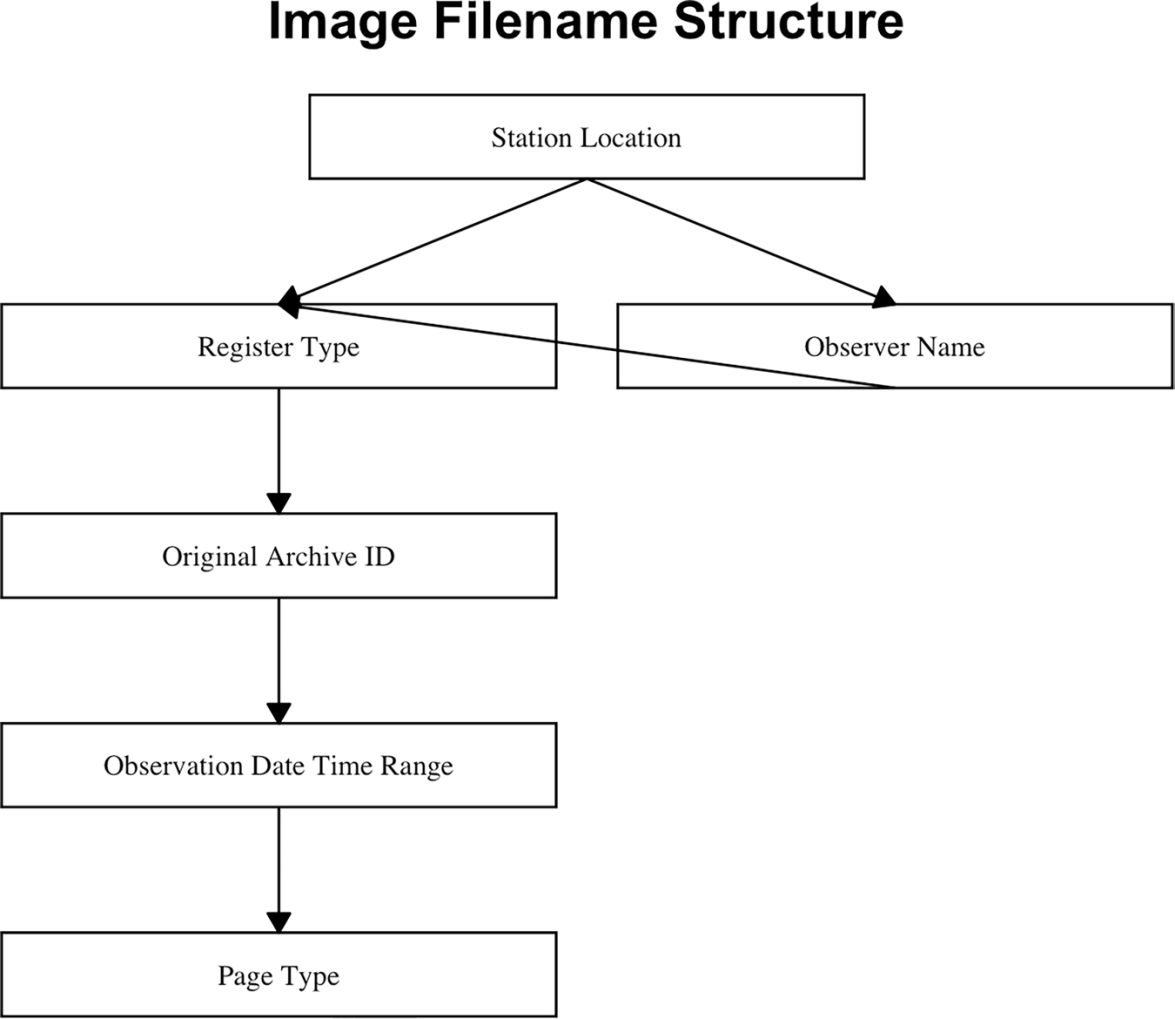
Image file nomenclature for a traceable data cycle process.

The image files obtained from the NARA M1958 repository^13^ were organized by station location and renamed according to register type, page type and date. Three considerations went into naming the image files. First, we wished to embed metadata of interest, such as location and time period covered by the data on the image files, to make it easy to locate the image file when at a later stage in the project weather data obtained from the image file were examined. Second, we wished to create a unique identifier for each image file. Finally, we wanted to maintain traceability of data from the original archive identifier to the data export in flat files. To accomplish these three goals, the elements listed in Fig. 5 were combined to form each image file name.

Each image file was given a unique identifier composed of the station location, the observer’s name if necessary for disambiguation, the register type, the originating archive identifier, the date of the observations and the page type. A typical file name is YorkFactory_USSS-316_M1958_1883-06-01_OBS-1.jpg. The image files were then uploaded to the web app, and appropriate transcription environments created to replicate each register type.

Images files were examined for quality and notes made on quality issues. Notes include comments on the condition of the image or of the original pages, such as “major ink smear”, “badly scanned, but mostly legible”, “pasted in values”, or “heavy bleedthrough.” The image files from NARA and NCEI were obtained from microfilm images of the original documents, so are at several removes from the original document (Fig. 2). In some cases, the original documents were not accessible to, due to either their fragility or their location in an overseas archive. Problems with the image files originated from two main sources. The first, which is inherent to the original document, was the quality of the original documents and conditions under which the weather observations were recorded and transmitted to the Smithsonian Institute. Tears in the pages were common in older documents and in pages from trading posts in Canada’s interior such as Fort Simpson or Michipocoton, which presumably had long postal journeys over rough terrain. A substantial number of pages had ink blots or bleedthrough obscuring sections of the page. At Wolfville, two months of humidity observations had recalculated values pasted over the originally recorded values. Some of these issues, particularly the bleedthrough and pasted sections, may have been easier to resolve if the images were available in a colour format, rather than black and white microfilm. The second major quality issue occurred when part of the document is obscured what appears to be tape or bindings on the document itself and possible microfilm issues. These problems can lead to irrecoverable portions of the observations on the page.

The images from UKMO were taken directly from the original sources, either by photograph or by high-quality scan. The photographs had issues with the page binding of the original document, making the values towards the edges of the pages where they were bound into a volume difficult to read and distorted. The images scanned professionally by the UKMO library and archives staff were the highest quality images with few issues.

### The register types

As most the records were inspired by the Smithsonian volunteer weather observing network, the observers recorded their observations on pre-printed forms and distributed first by the Smithsonian Institute (Fig. 2), and later by its various successors such as the US Signal Service. Similarly, the observations from the UKMO archives were largely taken by military observers with standard printed forms. Although an advantage of the printed forms is that they provide uniformity across stations and time, there is nonetheless some variety in forms. Formats changed over time as new observing variables were added or removed, or observing instructions were updated with evolving needs and improving instrumentation. Different forms were sent to different observers depending on the types of observations made. The forms were catalogued and given code based on the number of pages in the form: the “100” code family denotes a one-page form, “200” a two-page form, and so on (Table 1). A new register type was coded if the variables recorded changed or if the layout of the printed form changed. A subtype was noted if the observer added handwritten modifications or additional observations. This structure is designed to be flexible as new register types are continually being identified with new source materials.

**Table 1:** Register and Page Types.

Register Type	Code	Linked Page Types
UKMO Army Medical Department Form 848	AMD-848	OBS-F
	MRQ0-101	OBS-F
UKMO Royal Engineers 101	RE-101	OBS-F
UKMO Royal Engineers 102	RE-102	OBS-F
UKMO Royal Engineers 103	RE-103	OBS-F
UKMO Royal Engineers 104	RE-104	OBS-F
Register of Meteorological Observations 100	RMO-100	OBS-F
Toronto Magnetical Observatory	TMO-100	OBS-1, OBS-2
Upper Canada Grammar Schools	UCGS-200	OBS-C, OBS-D
United States Surgeon General	USSG-131	OBS-F
United States Surgeon General	USSG-102	OBS-F
US Smithsonian Institute 101	USSI-101	OBS-F
US Smithsonian Institute 201	USSI-201	OBS-F, CP-I, OBS-L, OBS-R, CP
US Smithsonian Institute 202	USSI-202	OBS-F, CP
US Smithsonian Institute 203	USSI-203	OBS-L, OBS-R
US Smithsonian Institute 204	USSI-202	OBS-F, CP
US Smithsonian Institute 206	USSI-204	OBS-F, CP
US Smithsonian Institute 206	USSI-207	OBS-F
US Smithsonian Institute 401	USSI-401	OBS-L, OBS-R, CP, EXT, SUM
US Smithsonian Institute 402	USSI-402	OBS-L, OBS-R, CP, INS
US Signal Service 314	USSS-314	OBS-1, OBS-2, CP
US Signal Service 316	USSS-316	OBS-1, OBS-2, CP
US Signal Service 412	USSS-412	OBS-L, OBS-R, CP
Custom page types (9)	CUS-XXX	various
UKMO War Office 848	WO-848	OBS-F

Some of the observations are recorded in personal diaries or in handwritten tables. These are unique, and as such do not conform to register page type cataloguing. These are given register types with the abbreviation of their location followed by a numerical designation for each change in the variables or layout of the diary or table. For example, the Amherstburg register^14^ changed formats several times, and the designations assigned to the register types are Amherstburg_AM-1, Amherstburg_AM-2, and Amherstburg_AM-3.

### The page type

Each register type can have one or more page types. A page type has a specific organization of information, both meteorological observations and metadata such station location, observer, date and variables observed. It should be noted that not all observers had sufficient time or the necessary instruments to record all variables listed in the forms, thus not all variables listed on a register type were necessarily recorded. The Smithsonian and later US Army Signal Service observation forms were sheets designed to be folded and sent by mail^13^. Their format changed over time: at times the form consisted of one sheet folded in two, with instructions printed on the reverse side. Later, the form consisted of four pages: a page of instructions, two inside pages for observations, and page for recording remarks and casual phenomena. The page types are divided into observations pages (OBS: Fig. 2a,b), casual phenomena pages (CP; Fig. 2c), and instruction pages (INS; Fig. 2d). On some forms the casual phenomena and instructions appear on the same page (CP-I).

Each page and register type have a specific combination of observing time and meteorological variables recorded. An example of the meteorological variables and the original measurements units for Register Type USSS 316 is shown in Table 2, along with modern equivalents and units where possible.

**Table 2:** Example of Meteorological variables for Register types USSS-316 with units and abbreviations: barometer, thermometer, cloud and wind, precipitation, humidty and weather remarks.

Variable grouping (Field group)	Variables observed (Fields)	Modern equivalent	Original unit	SI unit	Abbreviation
**Barometer**	Barometer Observed	Station pressure	inHg	hPa	p
	Barometer Observed Daily Mean	Station pressure mean	inHg	hPa	p_mean
	Barometer Attached Thermometer	Barometer Attached Thermometer	°F	°C	atb
	Barometer Correction and reduction	—	—	—	—
	Barometer Corrected for Temperature	Barometer Corrected	inHg	hPa	pcor
	Barometer Corrected Mean		in Hg	hPa	pcor_mean
	Mean Sea Level Pressure	Mean Sea Level Pressure	inHg	hPa	mslp
**Thermometer**	Thermometer Open Air	Air temperature	°F	°C	ta
	Thermometer Open Air Corrected	Air temperature corrected for index error	°F	°C	ta_cor
	Thermometer Open Air Daily Mean	Air temperature mean	°F	°C	ta_mean
	Thermometer Open Air Daily Range	Air temperature range	°F	°C	ta_range
	Minimum temperature	Minimum temperature	°F	°C	Tn
	Minimum Temperature Corrected	Minimum temperature corrected for index error	°F	°C	Tn_cor
	Maximum Temperature	Maximum temperature	°F	°C	Tx
	Maximum Temperature Corrected	Maximum temperature corrected for index error	°F	°C	Tx_cor
	Min Thermometer on Grass	Grass thermometer	°F	°C	Tng
	Thermometer in Sun	Blackbulb Thermometer	°F	°C	Txs
**Cloud**	Cloud Amount	Cloud cover	tenths	oktas	n
	Lower Cloud Amount	Lower Cloud cover	tenths	oktas	n
	Upper Cloud Amount	Upper Cloud cover	tenths	oktas	nh
	Cloud Kind	Cloud type	Abbreviated cloud name	International cloud atlas code	c
	Cloud Kind Lower	Cloud type lower	Abbreviated cloud name	International cloud atlas code	cl
	Cloud Kind Upper	Cloud type upper	Abbreviated cloud name	International cloud atlas code	ch
	Cloud Direction	Cloud direction	Cardinal direction	Compass degrees	cd
	Upper Clouds Course/ Direction	—	Cardinal direction	Compass degrees	hd
	Cloud Velocity		scale	scale	cv
	Upper Cloud Velocity		scale	scale	hv
**Wind**	Wind Direction	Wind direction	Cardinal direction	Compass degrees	dd
	Wind Force	Wind speed	Smithsonian scale (Sm)	m/s	w
	Wind Force	Wind speed	Beaufort scale (Bf)	m/s	w
	Wind Force	Wind speed	Pounds per square inch (lbs/ft^2^)	m/s	w
	Wind Velocity	Wind speed	Miles per hour (mi/hr)	m/s	w
	Wind Velocity Mean	Wind speed mean	Miles per hour (mi/hr)	m/s	w_mean
	Anemometer	Wind speed	Miles per hour (mi/hr)	m/s	w_anem
	Anemometer 24 hours	Wind distance run in 24 hours	Miles	m	w_anem_24
**Precipitation**	Precipitation (Rain Snow) Time Begin	—	Hour:minute	—	ptb
	Rain Time Begin	—	Hour:minute	—	rtb
	Snow Time Begin	—	Hour:minute	—	stb
	Precipitation (Rain Snow) Time End	—	Hour:minute	—	pte
	Rain Time End	—	Hour:minute	—	rte
	Snow Time End	—	Hour:minute	—	ste
	Precipitation Duration	—	Hour:minute	—	pr_dur
	Rain Duration	—	Hour:minute	—	rr_dur
	Snowfall Duration	—	Hour:minute	—	sd_dur
	Precipitation Type	Precipitation Type	text	text	pr_type
	Precipitation (Rain Snow) Amount	Precipitation amount	Inches	Rain: mm Snow: mm	pr
	Rain amount	Rain	Inches	mm	pr
	Depth Snow	Depth of snow	inches	cm	sd
	Snow Equivalent	Snow water equivalent	inches	cm	swe
**Humidity**	Psychrometer Dry Bulb	Dry bulb	°F	°C	tdb
	Psychrometer Dry Bulb Corrected	Dry bulb corrected	°F	°C	tdb_cor
	Psychrometer Dry Bulb Daily Mean	Dry bulb mean	°F	°C	tdb_mean
	Psychrometer Wet Bulb	Wet bulb	°F	°C	tb
	Psychrometer Wet Bulb Corrected	Wet bulb corrected for index error	°F	°C	tb_cor
	Difference wet-dry bulb	Difference wet-dry bulb	°F	°C	diff
	Wet Bulb Minimum	Wet bulb Minimum	°F	°C	tbn
	Wet Bulb Maximum	Wet bulb Maximum	°F	°C	tbx
	Relative Humidity	Relative humidity	%	%	rh
	Relative Humidity Daily mean	Relative humidity mean	%	%	rh_mean
	Dew Point	Dewpoint temperature	°F	°C	td
	Dew Point Daily Range	Dewpoint temperature range	°F	°C	td_range
Vapour	Vapour Tension/Force of Vapour	Vapour pressure	inHg	hPa	e
	Force of Vapour Mean	Vapour pressure mean	inHg	hPa	e_mean
Evaporation	Evaporation	Evaporation	in	mm	eee
**Remarks**	Weather	Manual Observation	Synoptic weather (manual observation mno)	Synoptic code	ww
	Remarks	Manual Observation	Synoptic weather (manual observation mno)	Synoptic code	ww
	Daily Weather Remarks/Average Weather	Manual Observation	Synoptic weather (manual observation mno)	Synoptic code	ww
	Phenomena at Observation	Casual Phenomena	Synoptic weather (manual observation mno)	Synoptic code	ww
	Casual Phenomena	Casual Phenomena	Synoptic weather (manual observation mno)	Synoptic code	ww
	Weather Since Last Observation	Manual Observation	Synoptic weather (manual observation mno)	Synoptic code	w1
	Phenomena Since Last Observation	Manual Observation	Synoptic weather (manual observation mno)	Synoptic code	w1
	aurora	—			au

The microfilming process of the original documents led to some documents being photographed as one image, but at other times be split into two separate images, a left-hand side of one original document page and a right-hand side another original document page. In order to capture this diversity of formats, the observations pages are subdivided into full observations pages (OBS-F), left-side pages (OBS-L) and right-side pages (OBS-R). The register types for the US Signal Service 314 and 316 had distinct pages, so these were named 1 and 2 rather than left and right (Fig. 2a,b). Each register type has one or more page type associated to it (Table 1).

### The transcription process

As observations are transcribed into the web app, they are saved directly into a database. Both our transcription environment and our data output are designed to resemble the original observations as closely as possible, for reasons of error reduction and conservation of scientific heritage.

**Fig. 6:**
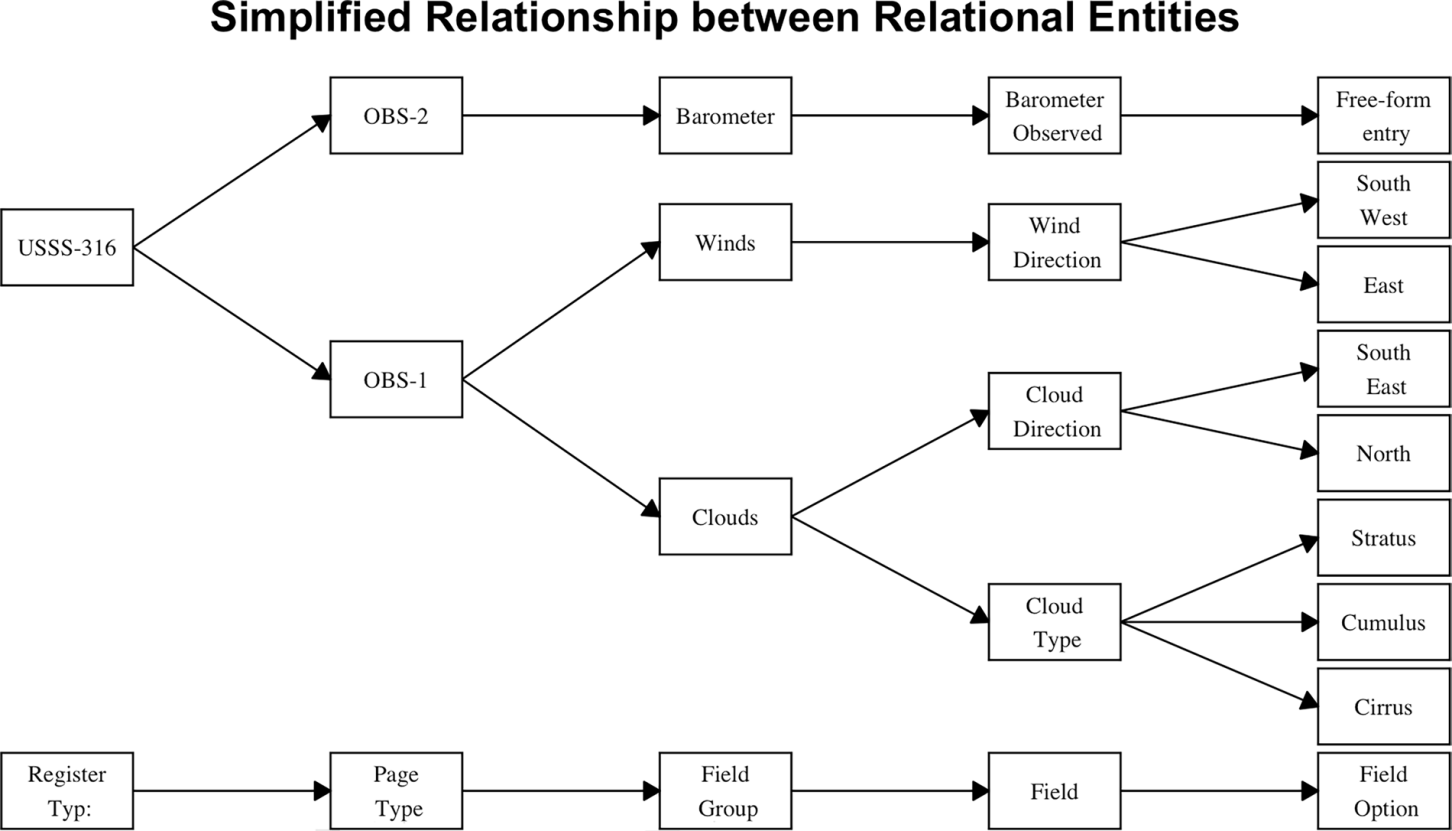
Example of the relationship between the register types, page types, field groups, fields and field options.

**Fig. 7:**
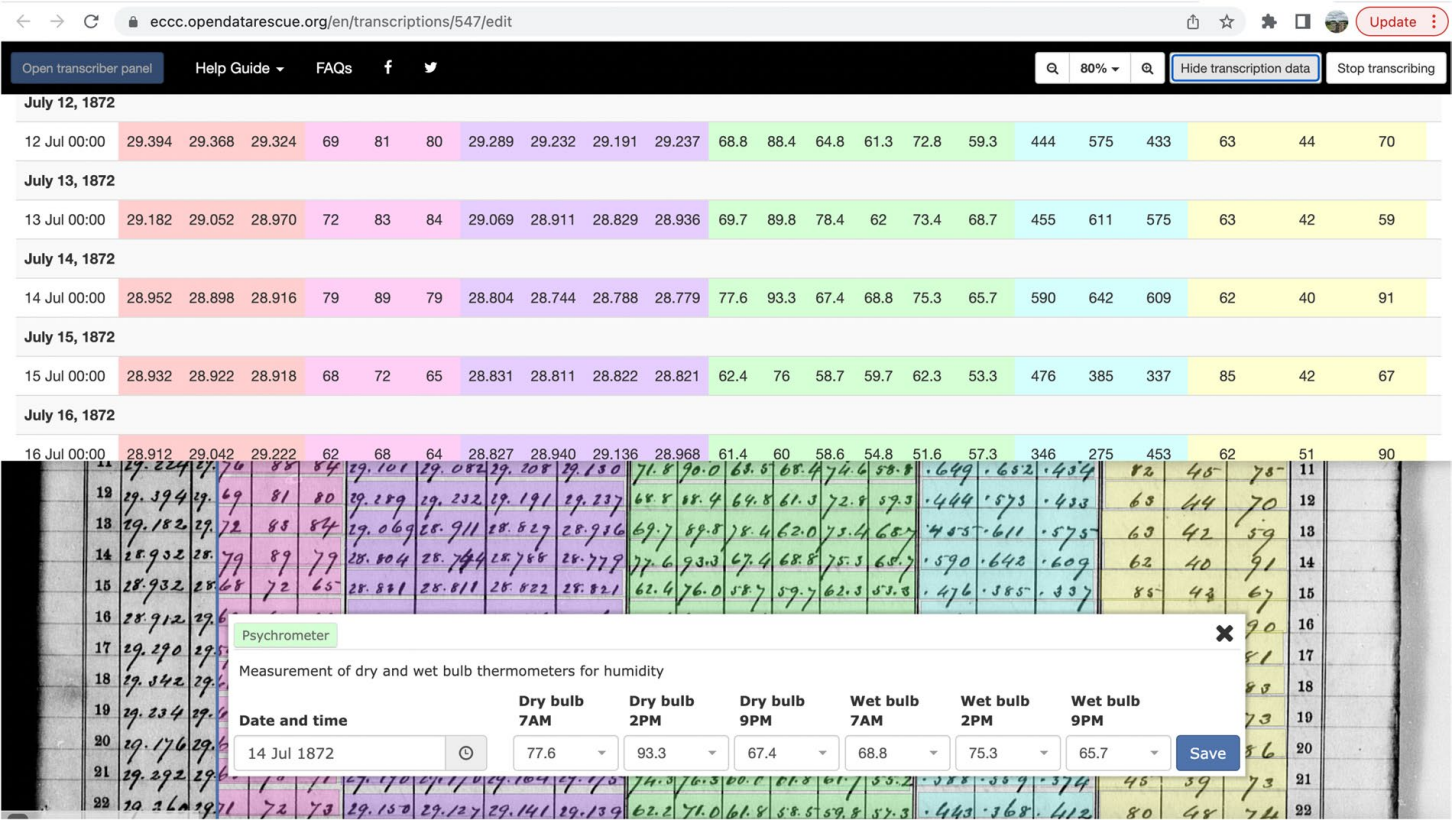
Example of the transcription app (https://eccc.opendatarescue.org/). Values from the image file (background) of the original weather register are entered into the transcription bar. The values are then saved directly into the database and can be verified by looking at the transcription data. Each field group is colour coded.

The transcription interface for each register type is therefore built up to reflect the observation groupings in the original register pages. Within the user interface (UI) on the administrator pages, a field group is created and named “Clouds”. Fields are created and named “Cloud direction”, “Cloud amount” and “Cloud kind” (Fig. 6). Field values are then created for the field “Cloud kind” which include options for the drop-down menu such as Cumulus, Nimbus, etc. These field values are then linked with the field “Cloud kind” in the UI (Fig. 7), the field “Cloud kind” is linked to the field group “Clouds”, and the field group clouds is linked to a register schema, such as USSS-316.

All linkages made in the UI are reflected in the back-end database. Fields values, fields and field groups can be used in more than one register schema. The field options for variables that have technically limited values, such as cloud type or wind direction, are accessed by a drop-down menu to limit transcription or interpretation errors (Fig. 7). Fields that are not constrained to limited options, such as barometer observations, have a free-text entry field.

Occasionally, modifications by the original observers necessitated the addition of new fields and sometimes even the creation of new register types during the transcription process. The observer for York Factory, for example, added minimum and maximum thermometer and supplementary barometer observations to the register. These additional observations, as well as additions to the printed observation forms, account for the differences between register types USSG-314 and USSG-316.

### Data transformation to modern standards

Table 2a–f gives an overview of the historical variables, the modern equivalents, historical and modern units, and the internationally agreed upon abbreviations for these variables where they appear in standardized filename datasets. Information on the specific observations, instruments and variables, such as the wind scale, are found in historical technical documents, such as *Instructions to Observers* pamphlets or articles^38,40^.

Not all historical variables have yet been given designated recognized modern equivalents. Most historical observations from the 19^th^ century are not recorded in standard SI units accepted in internationally exchanged data files (Table 2a–f). The information needs to be transformed into modern units as designated by, for example, the World Meteorological Organization (WMO) standards. Conversion values for pressure (Table 2a), temperature (Table 2b), precipitation (Table 2d), humidity (Table 2e) and precipitation are well-known. Wind and cloud directions are transformed from cardinal directions to degrees (Tables 2c, 3). Variables which are recorded in ordinal scales, such as wind force (Tables 2c, 4) or cloud velocity (Table 2c, are more difficult to transform into modern equivalents. The Smithsonian Institute developed a wind scale which was contemporaneous with, but not completely equivalent to, the Beaufort wind scale.

**Table 3:** Wind and cloud direction conversions from cardinal and intercardinal directions to degrees.

Cardinal direction	Abbreviation	Degrees (°)
North	N	0
North North East	NNE	22.5
North East	NE	45
East North East	ENE	67.5
East	E	90
East South East	ESE	112.5
South East	SE	135
South South East	SSE	157.5
South	S	180
South South West	SSW	202.5
South West	SW	225
West South West	WSW	247.5
West	W	270
West North West	WNW	292.5
NorthWest	NW	315
North North West	NNW	337.5
Calm/ Zero/Empty/ not perceptible	Calm	Calm
Variable	Var	Variable

**Table 4:** Smithsonian and USSG Wind Scale conversions.

USSG scale	USSG description	Smithsonian scale	Smithsonian description	Smithsonian range Miles/ hour	Eqv Beaufort	Eqv knots	Eqv m/s
0	calm						
1	Very gentle breeze	1	Very light breeze	2	1	1.7	1
2	Gentle breeze	2	Gentle breeze	4	2	3.5	2
3	Fresh breeze	3	Fresh breeze	12	3	10	5
4	Strong wind	4	Strong breeze	25	5	21	10
5	Very strong wind	5	High wind	35	7	30	15
6	Violent storm	6	Gale	45	8	40	20
		7	Strong gale	60	9	52	25
		8	Violent gale	75	11	65	35
		9	Hurricane	90	12	78	40
		10	Most violent hurricane	100	—	87	45

**Fig. 8:**
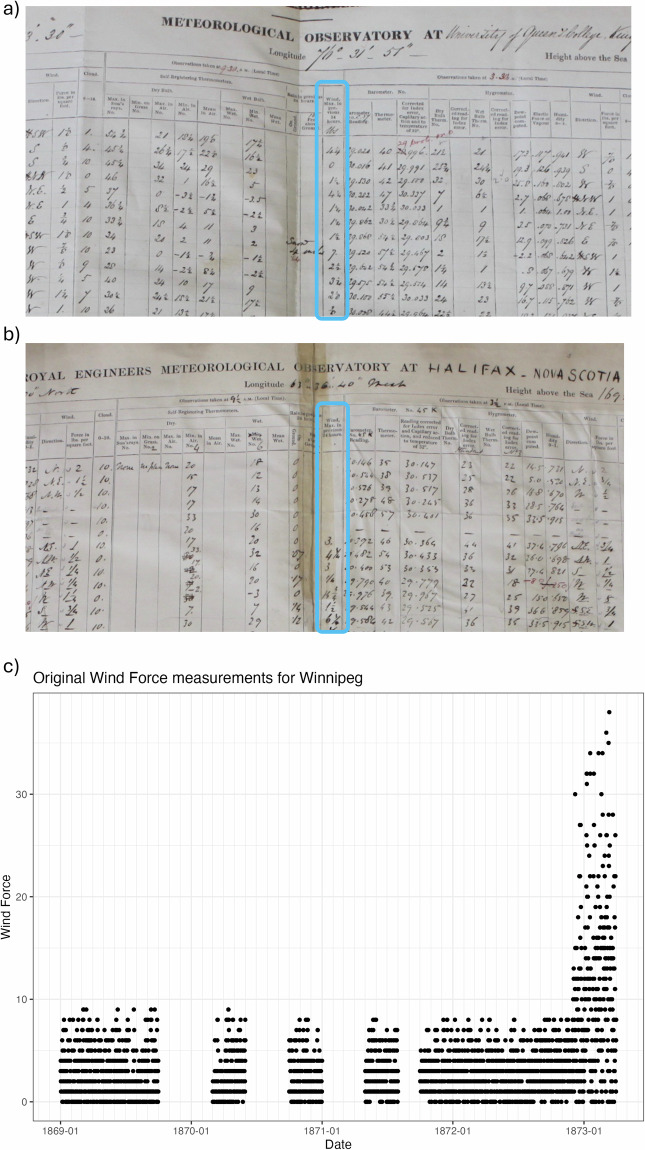
(**a**) Wind force measurements taken by the Royal Engineers at Halifax, in pounds per square inch, measured in pounds, ounces, and fractions of ounces; (**b**) wind force measurements by the Royal Engineers in Kingston, in pounds and decimal tenths of pounds, (**c**) original “wind force” measurements for Winnipeg, where an abrupt transition takes place from a one to ten scale (Smithsonian scale) to values up to the high 30 s.

The Royal Engineers were requested to measure the wind force in pounds per square foot. These were sometimes recorded in pounds and ounces, such as at Halifax (e.g. 3 15; Fig. 8a), and others in decimal pounds such as at Kingston (e.g. 3.8, Fig. 8b). At still other stations, such as New Westminster, the engineers measured the wind force using the Beaufort scale. Some of the wind force observations are further complicated by observers changing methods of recording partway through their records (e.g. from Beaufort to miles per hour; see Fig. 8c). With up to five different methods of recording, the wind force field is one of the most difficult to interpret correctly. As the SEF file formatting standard requests wind speed in m/s rather than wind force scales, wind force was one of the most complicated variables to address.

Most conversions were applied using the standard functions contained in the lmrlib.py routine produced by the International Comprehensive Ocean-Atmosphere Data Set (ICOADS) from NOAA^41^. The formula used for converting pounds per square inch to meters per second is given by Equation 1:

Equation 1. Conversion from wind force measured in pounds per square inch to metres per second

$${\boldsymbol{ws}}={\bf{0.44704}}\left(\sqrt{\frac{{\boldsymbol{wf}}}{{\bf{0.00256}}}}\right);$$

where ws is wind speed in m/s and wf is wind force in lbs/in^2^

Cloud velocities are also recorded in a scale of 1 to 10 (Tabe 2c). Cloud amounts, or cloud cover, were commonly recorded in tenths in the 19^th^ century, whereas the units prescribed by the SEF standards are octets. Cloud types used in the Smithsonian and other records are listed in Table 5, along with equivalents from the International Cloud Atlas^42^.

**Table 5:** Cloud types used in the Smithsonian registers and International Cloud Atlas equivalents.

Cloud kind in registers	description	International cloud Atlas code	Lower cloud kind in registers	description	International cloud Atlas code
S, St	Stratus	C_h_8	S, St	Stratus	C_l_6
C; Cir	Cirrus	C_h_6	C; Cir	Cirrus	C_h_6
N; Nim	Nimbus	C_m_2	N; Nim	Nimbus	C_l_7
C.s; Cir.st.	Cirro-stratus	C_h_8	Cu. S. Cu.st.	Cumulo-stratus	C_l_4
Cu.S.; Cu.st	Cumulo-stratus	C_m_7	C.s. Cir.St	Cirro-stratus	C_h_8
C.cu; Cir.cu	Cirro-cumulus	C_h_9	C. cu.; Cir. Cu	Cirro-cumulus	C_h_9
Cu	Cumulus	C_h_9	Cu.	Cumulus	C_l_2
P	Passing	C_m_4		Passing	C_l_2
Scud; Scudding		C_m_4	Scud; Scudding		C_l_5
Hidden^2^	(Haze, Smoke, Fog, Mist, lower clouds)	C_h_/	Hidden	(Haze, Smoke, Fog, Mist, lower clouds)	C_l_/
None; Clear	Zero, 0	C_h_0	None; Clear	Zero, 0	C_l_0

Note: subscripts not possible in csv or SEF files.

^1^Hidden clouds are given cloud amount code 9.

Historical weather remarks are more difficult to translate to modern synoptic weather codes (Tables 2f, 6). The relationship between the weather conditions described in the historical registers and the modern Canadian synoptic is not fully equivalent. There are conditions described in the historical documents which have no parallel in the synoptic codes and similarly, some synoptic codes which will have no exact equivalent in the historical wording of past weather conditions.

**Table 6:** Historical Weather remarks and equivalent Canadian Synoptic Weather Codes^46^.

Weather descriptor	Synoptic code
snow	SN
rain	RA
thunderstorm	TS
lightning	LT
freezing rain	FZRA
freezing drizzle	FZDR
Ice crystals	IC
fog	FG
freezing fog	FZFG
mist	BR
fog patches	BCFG
shallow fog	MiFG
blowing snow	BLSN
blowing dust	BLDU
drifting snow	DRSN
hail	GR
small hailstones	SHGS
drizzle	DZ
shower	SH
squalls	SQ
smoke	FU
haze	HZ
dust haze	DY
sleet (historical only; not in modern synoptic code)	IP + RA + SN

## Data Record

The dataset is available at the US NOAA National Centers for Environmental Information (NCEI)^43^. The dataset is titled AIR TEMPERATURE, Surface pressure, and others collected from FIXED STATIONS OF CANADA in Canada from 17680911 to 18840229, with the NCEI Accession Number 0304217. The data can be found at 10.25921/g637-9093.

### Metadata

The metadata standard used here builds on the ISO 11905 standards for geographical information, the WIGOS (World Meteorological Organization Integrated Global Observing System) recommendations^44^ and the extensions and recommendations of the Copernicus Working Group Best Practice Guidelines^45^. Further modifications have been made here to adapt to the contingencies of historical climate information. We include historical location designators such as historical latitude, historical longitude and additional historical location designators to reflect the fluidity and nomenclature of Canadian territorial designators.

### Data Export files 1: CSV files

After the technical validation (see next section), csv files are produced whose aim is to replicate the original observations as closely as possible in the original units. This is to provide a machine-readable reproduction of the historical records. The files are produced by register type, as each register type reflects differences in the times of observation or variables observed. The csv files for York Factory are thus YorkFactory_USSI-412_1874-10_1876-09.csv, YorkFactory_USSI-314_1876-12_1881-12.csv and YorkFactory_USSI-316_1882-01_1884-02.csv.

### Data Export Files 2: SEF files

The export format of the data files is based on the station exchange format (SEF) developed by the Copernicus Working Group. The filename is constructed using the elements of the originating data project source or repository, the station name, the start date, the end date, and the variable abbreviation.

The first eleven lines of the SEF file contain standardized metadata, comprising the SEF version, the station ID, the station name, the latitude, the longitude (degrees east, station altitude, source, data link, variable abbreviation, temporal statistic and measurement unit. The twelfth line describes the structure of any metadata included in the subsequent data lines, as well as any metadata included in the overall data series (for example, “UTCOffset = YES” specifics that the dates and times in the file have been transformed to the Universal Time Coordinate).

### Data Export Files 3: Final SEF and NCEI format csv

The final set of SEF files are compiled once the post-processing is complete. As the database is updated with a final ISO standard table, csv files according to NCEI specifications, with station metadata, observation metadata, and observations for all variables for each station in a single csv file.

## Technical Validation

### Step 1: Page checks

The first validation step is a visual check by quality-control specialists on the transcribed data through the transcription app. The data table is compared to the uploaded image file, and visual inspection for missing rows of data indicating mis-entered dates, repeated rows of data, and other common transcriptions errors that are difficult or time-consuming to verify without recourse to the original document were prioritized during this initial check. These include common historical meteorological shortcuts such as use of ditto marks to indicate repeated values or omission of leading digits before the decimal. A first check of illegible values, due to document degradation, microfilming or imaging issues, or handwriting issues is also performed at this stage.

All issues are noted in a log file, though not all logged issues are necessarily errors. Many are notes made by transcribers on the comments, annotations or inconsistencies, such as changes in observation times made by the original observers. Common transcription errors that are difficult to correct automatically were found to be writing 57 for 51, due to mistaking the bar in the handwritten five for a bar in the digit seven; misplacing a decimal, such as typing 1.01 instead of 10.1, and forgetting to enter the correct date.

### Step 2

The second validation step is performed in a program external to the app. Each entry for a given station is extracted from the database. If more than one transcription exists for a station ID, variable and date the most recently updated value is selected.

Entries are then scanned for values which equate to no data, including “none”, “missing”, “retracted”, “empty”, and other transcription equivalents such as dashes, spaces, and null values. These are coded to −999 and the quality control flag is set to missing. Values entered as “illegible” or with other error codes are set to −999 with appropriate flags (Table 7). Many transcription errors are noted automatically at this stage.

**Table 7:** Validity codes.

Data issue	Examples	Value code	flag
Missing	Blank, “, -, “not taken”, “none”,”suspended”,”absent on duty”	−999	“missing”
Instrument error	“out of order”, “broken”, “unserviceable”, “covered with snow”, “not reliable”	−999	“Instrument error”
illegible	Ink blot, page tear, microfilm cut-off	−999	“illegible”

The validation then proceeds depending on the variable type, with different procedures depending on the assigned units. For all variables with units of inches of mercury, degrees Fahrenheit or percentage, values are checked for common transcription issues such as double decimal points, commas instead of periods for decimals or spaces in the number. These are automatically corrected in the code and an error message with a structured query language (sql) correction code is written to a log file for each variable. Values for fields such as temperatures, pressures or precipitations should also all be decimals, so they are transformed into floats. Observers sometimes recorded values as fractions, which then must be transformed into decimal values.

The observers often did not note the leading zero and decimal when recording vapour pressure. Vapour pressure values are tested for range and divided by 100 if no decimal point is detected and the values are greater than 1. Similarly, some observers did not record the leading digits before the decimal for barometric pressure. In most cases either the transcribers added the leading digits based on previous barometric pressure values, or they were added at the first validation stage, but these values needed to be corrected more often than completed written observation in the third validation stage. Common issues and solutions are listed in Table 8.

**Table 8:** Transcription validation issues.

Data type	Validation issue	Solution	flag
Float number (pressure, temperature, precipitation, percentage) Precipitation, temperature	Space instead of decimal point	Replace with single decimal	
	Comma instead of decimal point	Replace with single decimal	
	Two decimal points	Replace with single decimal	
	+ or space before value if in original document	Remove extraneous character	
	Double negative	Remove second negative	
	fraction	Convert to decimal	
Vapour pressure	Missing decimal	Multiply by 100	multiply by 100
All fields	Multiple entries in fields	Separate entries	
Precipitation	Multiple entries in precipitation fields	Sum entries	Two values added
Wind, cloud	Multiple entries	Split entries	
Precipitation	“Trace”, “slight” or “in” [inappreciable] in precipitation field	Replace with 0.005 (inches)	trace
Cloud	Cloud amount “Hidden”,” Fog”,”Haze”,”Mist” or “Smoke”	Cloud amount = 9	

Variable specific validations are noted in Table 9. Observers often wrote specific symbols for recurring, site-specific situations. For example, difficulties occurred in recording humidity levels occurred at York Factory when the air temperature was less than −20 °F (−28.9 °C), as the tables used for calculating humidity did not extend past −20 °F. There may have been additional problems with the reliability of the calibrations of the dry and wet bulb thermometers below this temperature. The observer recorded this with a modified asterisk symbol.

**Table 9:** Out of range flags.

Variable	Range (original units)	SI units	flag
Barometric pressure	<27 inHg	914.2 hPa	out of range
	<28.5 inHg	965.0 hPa	low value
	>30.6 inHg	1036.1 hPa	High value
	>32 inHg	1083.5 hPa	out of range
Vapour pressure	<0 inHg	0 hPa	out of range
	>2 inHg	6.8 hPa	out of range
Temperature	<−50°F	−45.5 °C	out of range
	<−45°F	−42.7 °C	low value
	>100°F	37.8 °C	High value
	>120°F	48.9 °C	out of range
Relative humidity	<0%		out of range
	>100%		out of range
Wind force	<0		out of range
	>10		out of range
Cloud velocity	<0		out of range
	>10		out of range
Cloud amount	<0	<0	out of range
	>10	>8	out of range
Precipitation	<0		out of range
	>30 in	36.2 cm	out of range

There is the potential for bias in values such as cloud cover, wind or precipitation where the observers did not always record “zero” when there was no cloud, wind or precipitation, but instead left the entry blank. This makes disambiguating data that was not recorded from days without a particular phenomenon, such as wind, cloud amount or precipitation, difficult. Users are advised to keep in mind this occasional issue between “no phenomena to record” and “no observations were made”. We advise caution, and for users to consider the entirety of the observation, such as whether other observations are present for that observation time, and whether these or the weather remarks support an interpretation of “no phenomena to observe” when using these data.

### Range checks

Once the transcribed values were in a form appropriate for the variable they represented (float, integer, text, etc), the following value checks could be performed. Given the wide range and variability of the Canadian weather and climate, at this validation stage these checks are only at the extreme boundary edge of the expected range for the climate zone. If the values are not determined to be errors, they are flagged as “out-of-range” and left in the database. The range checks were performed on the original values before transformed into SI units. The ranges and flags for numerical values are listed in Table 9.

Values which are noted as out of range are checked to ensure there are no transcription errors such as a misplaced decimal.

Some of these concerns, such as multiple entries in the original handwritten entries, original entries containing questions marks, asterisks, or other non-standard characters, out-of-range values such as “11” for scale values with a maximum value of ten, are conserved in the original database and are only modified in post-processed files. On the other hand, when an observer made an error which is easily verified and corrected, such as writing “39.91” for a barometer entry rather than “29.91” when the sequence of barometric pressure shows a clearing falling pressure trend from 30 inches to 29 inches, the values are updated directly in the database. A data audit feature in the app tracks all changes made to the data post-transcription.

The final action in step 2 is the transformation to SI units and the production of the SEF files.

### Step 3

The third validation check is to verify the data produced in the SEF files. Values are verified by station in groups of similar variables, for example temperature files are verified together, pressure files are verified together, and so on. Large deviations are investigated for transcription errors such as transcribing as 37 instead of 73. By transcribing all the observations, it is possible to make use of the integration of the observations: for example, the open-air temperature and the dry bulb temperature should be very similar; the mean daily humidity should be arithmetically close to the sum of the individual humidity observations, corrected pressure should usually be greater than station pressure, and so on. Any large deviations from expected patterns of comparative values can be quicky investigated.

Other transcriptions issues include the inclusion of additional observations, observations which were not recorded in column specified, and systematic non-standard recording of observations. These required amending of the transcription platform and establishing new protocols to best capture the meteorological information in a systematic and complete manner. One example was the recording of minimum temperatures in the force of vapour fields. Another was the systematic recording of precipitation events in the cloud column by several observers, while noting the cloud cover as 10/10^th^. While not transcription errors, these deviations from standard practice on the part of the historical observers led to a modified protocol of adding the precipitation and weather notes into a newly added “Remarks” field, noting the type of precipitation in the precipitation type field, and marking the cloud type as “overcast”. This necessitated the substantial re-transcription of numerous pages. Overall, a total of approximately 5400 annotations (field groups) changed during the validation in the database. From this we arrive at a validation change rate of 2.4%.

### Final dataset

**Fig. 9:**
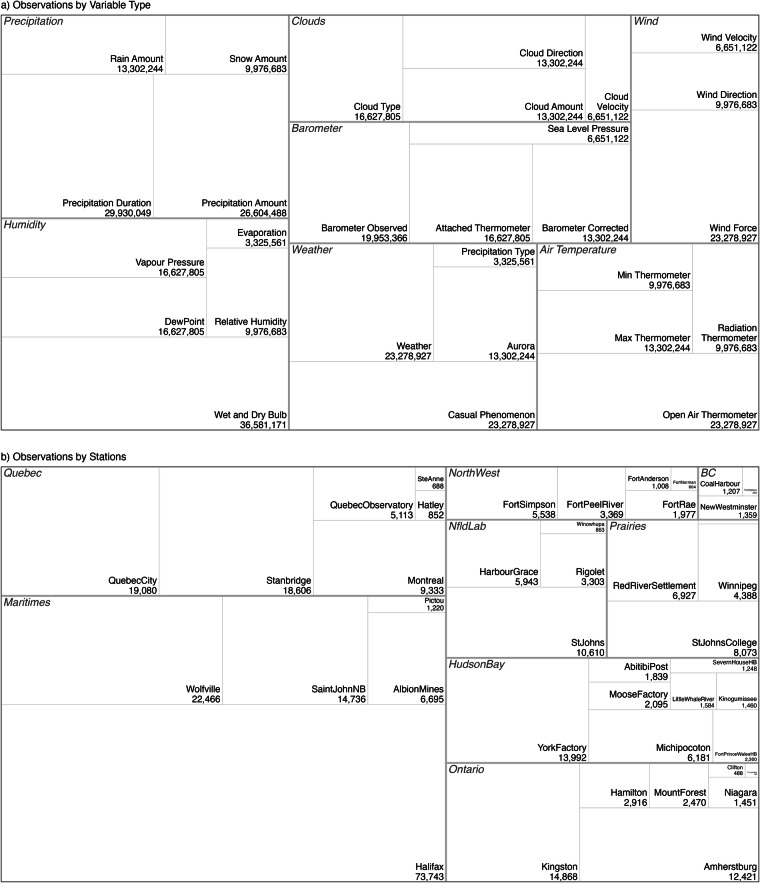
Overview of the dataset (**a**) by variable type and (**b**) by region and station.

Figure 9a shows the overall number of observations in each weather category for the dataset.

As can be seen in Fig. 9a, the observations that can by judged by eye, without the need for an instrument, are the most common, with wind direction and force, cloud type, direction of movement, and velocity forming a large part of the dataset. Thermometer observations are the next most common, as the thermometer is a reliable and robust instrument. Barometric pressure observations are nearly as plentiful as thermometer observations, while humidity measurements, mainly derived from dry and wet bulb thermometer observations, are also widely reported. Precipitation observations are not as common, partly since while wind and cloud (or their absence), and temperature, can be observed continually, precipitation is only observed when it occurs, which for most of the stations is typically about 10 days per month. Nevertheless, surprisingly few observers kept regular quantitative measurements of precipitation. This could be due to the prevalence of snowpack across the country and the relative difficulty of accurately measuring snow. Weather remarks were not kept in a regular fashion by all observers, with some providing detailed accounts of the weather and some none.

Observations become sparser to the west and north as can be seen in Fig. 9b. A large proportion of the observations are from Newfoundland, Labrador and the Maritimes; the provinces bordering on the Atlantic Ocean. Thanks to the efforts of observers connected to the Hudson Bay Company, there are also observations from the central and north-central parts of the country. There are fewer observations from the Northwest and Pacific Ocean regions.

**Fig. 10:**
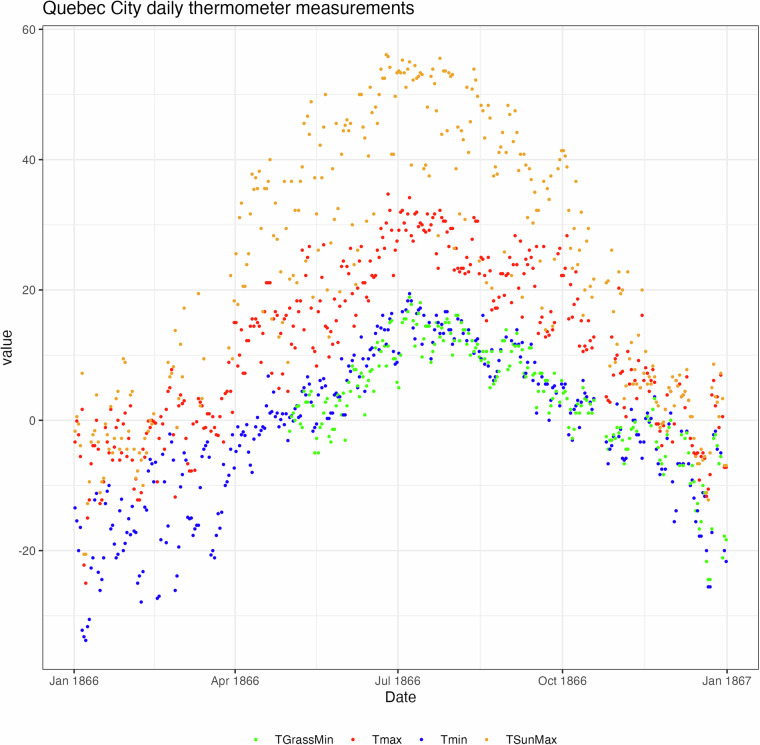
Grass minimum, minimum, maximum and blackbulb thermometer measurements shown for Quebec City, 1866.

Four separate measurements of daily temperature are shown in Fig. 10. The internal coherence of the data as a whole, and the use of inter-variable comparisons as data verification, are both expressed in this figure. The lowest thermometer readings are, as expected, the grass minimum thermometers (Fig. 10, green dots), as this measurement is designed to capture the outgoing longwave radiation emitted from the ground surface. The minimum air thermometer readings (blue) are the next lowest. Maximum air temperatures (red) are the second highest, with the blackbulb thermometer in the sun readings (yellow), designed to indicate the amount of incoming shortwave radiation, showing the highest values. If any value is unexpected relative to any others (for example, if the maximum air temperature is less than the minimum, or the blackbulb less than the air maximum), it can be investigated for errors.

**Fig. 11:**
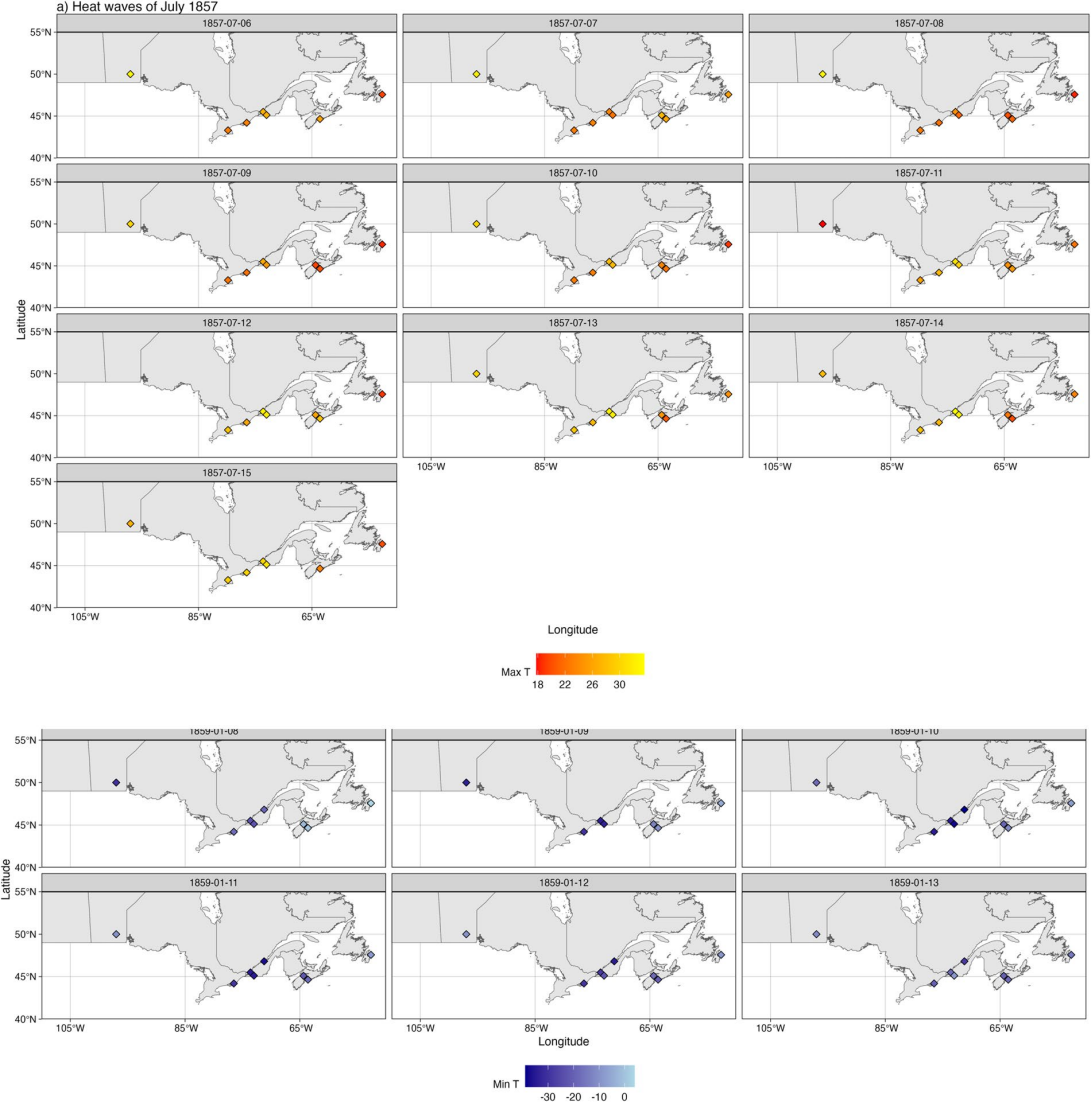
Examples of weather events from the data set. (**a**) A heat wave swept from west to east across southern Canada between July 6 and July 16 1857; (**b**) some of the coldest temperatures ever recorded in southern Canada were during the cold snap of January 8 to January 14 1859.

We can further examine specific weather revealed in this dataset by looking at cases of extreme temperature events: the heatwaves of July 1857 and the cold spell of January 1859 (Fig. 11).

At least two distinct heatwave events occurred in Canada in July 1857. The first, from July 6 to July 15, is shown in Fig. 11a. High temperatures first occurred in the Red River Settlement (Winnipeg) to the west over July 6 to July 8, while temperatures remained cool in the east. Warmer conditions started to develop over central Canada on July 10, while the west started to cool on July 11. Conditions continued warm in southern Ontario and Quebec until July 15. A second heatwave (not shown) again spread across the country from July 22 to July 28, this time with temperatures reaching the high 20 s to low 30 s °C in the Atlantic provinces.

Eighteen months later, in January 1859, some of the lowest temperatures ever recorded were experienced in southern Canada. Once again starting in the west, temperatures were below −33 °C at the Red River Settlement (Winnipeg) on January 9. Temperatures below −34 °C were recorded in Kingston on the 10^th^, −34 °C in Montreal on the 10^th^ and 11^th^, and −39 °C in Quebec City and −38 °C in Stanbridge on the 10^th^. By January 10th Red River had warmed to −17 °C, and to −10 °C om the 11^th^ as the cold wave swept eastward. The cold also moderated as it reached the Atlantic provinces, with a minimum of only −21 °C in Halifax on January 12 and −15 °C in St Johns on January 13.

## Usage Notes

The data here has not been homogenized or corrected from the original values beyond that described in the validation section.

The SEF conventions require times to be converted to the Universal Time Co-ordinate. Given the uncertainties in the longitude estimates in many of the historical documents, we have used modern time zone approximations rather than rely on the longitude estimates for time conversions. For fields such as “time precipitation began” and “time precipitation ended”, observers sometimes wrote vague indicators such as “afternoon”, “since this morning” or “overnight”, rather than precise times. Precipitation totals were sometimes also measured over event durations which could last several days, leading to some precipitation amounts that are higher than expected for single day measurements.

Observers also regularly noted problems with instruments due to external factors. The humidity readings at York Factory were on occasion unable to be recorded as the temperatures were too low to enable readings. The rain gauge on the campus of Acadia College at Wolfville was reported broken on several occasions.

The instruments used to measure humidity are of unknown quality and work remains to be done to investigate historical humidity measurements.

Other known issues include the pressure values for Winnipeg being too low for credibility from January to July 1869. Wolfville pressure is also lower between September 1855 and March 1856 than for the remainder of the record. The relative humidity values for Wolfville between January and May 1856 are also suspect; most of them are too high if considered as accurate recordings but too low if divided by 1000, assuming the observers did not write in the decimal point. The mean sea level pressure values for Mount Forest are sometimes out of range, being higher than could be reasonably expected. The pressure values for Halifax Royal Engineers (RE) suggests two different sites; one from August 1852 to December 1856, and a different site from September 1858 to March 1862. Pressure values for Halifax Dockyards (DY) are higher than physically possible for July and August 1860. These values have been removed from the archived dataset version 1.0 pending further investigation. The original values are available from the authors.

## Data Availability

The code used to run the transcription website is archived at https://github.com/open-data-rescue/climate-data-rescue. The code used to perform data validation checks and transform the values from the database of transcribed values to SI units and SEF standards is archived on GitHub at https://github.com/open-data-rescue/ODR-weather-data-files/tree/main/Canadian_stations/programs. The main source code is sef_generator_global.py. It has two possible execution modes: one to be run for each individual station with a json file with the station particulars, the other to access all the relevant metadata from the database and run all the stations in a loop. The code is designed to switch on parameters such as UTC offset or wind and cloud SI conversions, as full compliance with these standards can make validation more difficult at different stages of data production. The json files and metadata tables also have parameters to adjust for non-standard observation times or changes in observing practice, adjustable by variable, in the original observations. Further code modifications are made on a continual update basis as other non-standard or observer-based changes are discovered in the historical observation set. Code to read the data files can be found at https://github.com/open-data-rescue/ODR-weather-data-files/tree/main/Canadian_stations/programs.
